# Baseline antipsychotic prescription and short-term outcome indicators in individuals at clinical high-risk for psychosis: Findings from an Italian longitudinal study

**DOI:** 10.1192/j.eurpsy.2024.1588

**Published:** 2024-08-27

**Authors:** L. Pelizza, E. Leuci, E. Quattrone, M. Menchetti, A. Di Lisi, C. Ricci

**Affiliations:** ^1^Università di Bologna, Bologna; ^2^AUSL di Parma, Parma; ^3^Alma Mater Studiorum University of Bologna, Bologna, Italy

## Abstract

**Introduction:**

The prognostic prediction of outcomes in individuals at clinical high-risk for psychosis (CHR-P) is still a significant clinical challenge. Among multiple baseline variables of risk calculator models, the role of ongoing pharmacological medications has been partially neglected, despite meta-analytical evidence of higher risk of psychosis transition associated with baseline prescription exposure to antipsychotics (AP) in CHR-P individuals. In particular, baseline AP exposure in CHR-P individuals may be considered as a functional equivalent of the psychometric transition to psychosis, as already postulated in the original ‘Ultra High-Risk’ model.

**Objectives:**

The main aim of the current study was to test the hypothesis that ongoing AP need at baseline indexes a subgroup of CHR-P individuals with more severe psychopathology and worse prognostic trajectories along a 1-year follow-up period.

**Methods:**

This research was settled within the ‘Parma At-Risk Mental States’ program. Baseline and 1-year follow-up assessment included the Positive And Negative Syndrome Scale (PANSS) and the Global Assessment of Functioning (GAF). CHR-P individuals who were taking AP medications at entry were included in the CHR-P-AP+ subgroup. The remaining participants were grouped as CHR-P-AP-. The acquisition of drug and outcome information was collected both at baseline and across the follow-up period. Finally, logistic regression analyses with dichotomized 1-year outcome parameters (previously showing statistically significant differences in inter-group comparisons) as dependent measures and sociodemographic and clinical characteristics as independent variables were also performed.

**Results:**

Hundred and seventy-eight CHR-P individuals (aged 12–25 years) were enrolled (91 CHR-P-AP+, 87 CHR-P-AP-). Compared to CHR-P AP-, CHR-P AP+ individuals had older age, greater baseline PANSS ‘Positive Symptoms’ and ‘Negative Symptoms’ factor subscores and a lower GAF score. At the end of our follow-up, CHR-P-AP+ subjects showed higher rates of psychosis transition, new hospitalizations and urgent/non-planned visits compared to CHRP- AP- individuals.

**Conclusions:**

The current study suggests that AP need is a significant prognostic variable in cohorts of CHR-P individuals and should be included in the current risk calculators. In particular, the results of this study conducted in a realworld clinical setting indicate that the rate of CHR-P individuals who were already exposed to AP at the time of CHR-P status ascription was higher than those reported in recent meta-analyses on this topic. Moreover, our findings confirm that baseline AP prescription appears to increase psychotic transition risk.

**Disclosure of Interest:**

None Declared

